# Transcriptome and proteome analysis of tyrosine kinase inhibitor treated canine mast cell tumour cells identifies potentially kit signaling-dependent genes

**DOI:** 10.1186/1746-6148-8-96

**Published:** 2012-06-29

**Authors:** Robert Klopfleisch, Anja Meyer, Patricia Schlieben, Angelika Bondzio, Chris Weise, Dido Lenze, Michael Hummel, Ralf Einspanier, Achim D Gruber

**Affiliations:** 1 Department of Veterinary Pathology, Freie Universität Berlin, Robert-von-Ostertag-Strasse 15, Berlin, 14163, Germany; 2 Department of Veterinary Biochemistry, Berlin, Germany; 3 Institute of Chemistry/Biochemistry, Berlin, Germany; 4 Institute of Pathology, Charité Universitätsmedizin, Berlin, Germany

**Keywords:** KIT, Mast cell tumour, Dog, 2D-DIGE, MALDI, Mastocytosis, Tyrosine kinase inhibition

## Abstract

**Background:**

Canine mast cell tumour proliferation depends to a large extent on the activity of KIT, a tyrosine kinase receptor. Inhibitors of the KIT tyrosine kinase have recently been introduced and successfully applied as a therapeutic agent for this tumour type. However, little is known on the downstream target genes of this signaling pathway and molecular changes after inhibition.

**Results:**

Transcriptome analysis of the canine mast cell tumour cell line C2 treated for up to 72 hours with the tyrosine kinase inhibitor masitinib identified significant changes in the expression levels of approximately 3500 genes or 16% of the canine genome. Approximately 40% of these genes had increased mRNA expression levels including genes associated with the pro-proliferative pathways of B- and T-cell receptors, chemokine receptors, steroid hormone receptors and EPO-, RAS and MAP kinase signaling. Proteome analysis of C2 cells treated for 72 hours identified 24 proteins with changed expression levels, most of which being involved in gene transcription, e.g. EIA3, EIA4, TARDBP, protein folding, e.g. HSP90, UCHL3, PDIA3 and protection from oxidative stress, GSTT3, SELENBP1.

**Conclusions:**

Transcriptome and proteome analysis of neoplastic canine mast cells treated with masitinib confirmed the strong important and complex role of KIT in these cells. Approximately 16% of the total canine genome and thus the majority of the active genes were significantly transcriptionally regulated. Most of these changes were associated with reduced proliferation and metabolism of treated cells. Interestingly, several pro-proliferative pathways were up-regulated, which may represent attempts of masitinib treated cells to activate alternative pro-proliferative pathways. These pathways may contain hypothetical targets for a combination therapy with masitinib to further improve its therapeutic effect.

## Background

Canine mast cell tumours (MCT) are currently treated with one or a combination of four different therapeutic approaches: surgical, radiation, classical chemotherapy and the recently introduced tyrosine kinase inhibitors (TKI) [1-3]. The latter mainly act by inhibiting the stem cell factor receptor KIT, the activation of which is one of the most important proliferation stimuli of normal and neoplastic mast cells [1,4].

KIT is constitutively expressed on normal and neoplastic canine mast cells [5]. Due to its central role in mast cell biology and carcinogenesis a special research focus has been placed on the understanding of KIT for canine MCT development, malignant progression and treatment in recent years. Several KIT mutations have been identified, which are associated with aberrant KIT signalling and thus seems to be a major carcinogenic factor for a subset of canine mast cells [1,6]. For instance, tandem duplications in the juxtamembrane subunit of KIT have been identified in up to 12% of all canine MCT and 40% of malignant grade III MCT [6-8]. This mutation results in a constitutively activated KIT tyrosine kinase and leads to increased malignant behaviour in most affected tumours [1]. As a consequence TKI have been introduced for MCT treatment [9-12]. The action of TKI is not restricted to the tyrosine kinase subunit of KIT but other tyrosine kinases are also inhibited [12,13]. Nevertheless, the activity of non-KIT tyrosine kinases appear much lesser relevant for canine mast cell proliferation and the principle of TKI action in canine MCT is thus thought to mostly rely on KIT inhibition [1,13].

Masitinib is a tyrosine kinase inhibitor that selectively targets KIT, the platelet-derived growth factor receptors α and β (PDGFR-α/β) and the Src family kinases [13]. Masitinib has been successfully used in the treatment of canine MCT [9,14]. In addition to its direct effects on MCT by KIT inhibition, in vitro and in vivo, pilot studies indicate that masitinib also has a potential for chemosensitisation to classic chemotherapeutic agents including gemcitabine, vinblastine and doxorubicin [15-17].

Despite these ongoing efforts in KIT research in veterinary oncology, little is known on the downstream signal transduction pathways, target genes and cell functions associated with KIT activity. The present explorative study therefore aimed at identifying the transcriptional and translational changes after treatment of neoplastic canine mast cells with the TKI masitinib using transcriptome and proteome analysis.

## Results

### Cell proliferation, metabolism and death after masitinib treatment

Treatment of C2 cells with 100 nM masitinib induced a complete growth arrest with stable cell counts during 72 hours of treatment. In contrast, cell numbers in untreated C2 cells constantly increased during the experiment with an almost 3-fold rise of cell counts after 72 hours.

**Figure 1:**
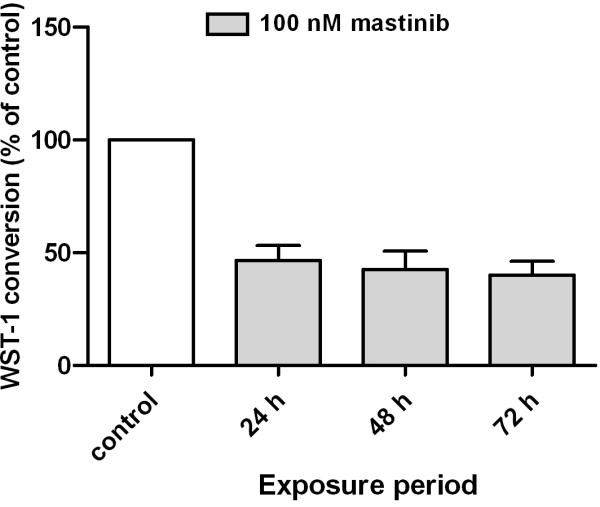
**Effect of masitinib on WST-1 conversion.** Masitinib treatment (100 nM) reduced the metabolic activity of C2 cells by 54% after 24 hours of treatment. The metabolic activity further decreased up to 40% at 72 hours. Data shown represent the means+/−SD of five independent experiments with C2 cells, each performed in three to five wells.

A WST-1 assay was performed to assess mitochondrial function before exposure, and after 24, 48 and 72 hours of masitinib exposure (Figure 1). There was an approximately 60% reduction in WST-1 conversion of masitinib treated cells at all indicated time points when compared to the initial activity of the untreated cells. A starting point analysis was chosen to reduce the influence of variable cell concentrations of treated and untreated cell cultures and the different metabolic activity during the different growth curve phases of untreated cells.

**Figure 2:**
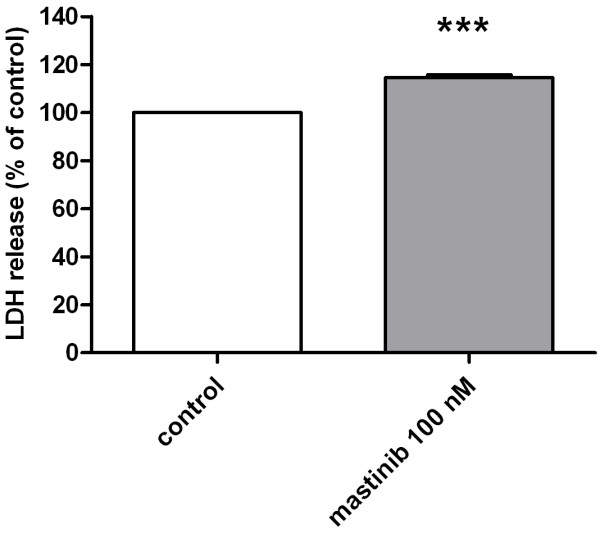
**Effect of masitinib on LDH-release.** Masitinib induced a slight increase in LDH release of C2 cells following 72 h treatment indicating only a mild cytotoxic effect of the masitinib concentration (100 nM) used. Data shown represent the means+/−SD of six independent experiments each performed in four to six wells.

LDH-leakage assay identified a significant increase of 11.4% after 72 hours but no changes after 24 and 48 hours, indicating only a reduced level of cell death and cytotoxicity at the masitinib concentration used (Figure 2).

### Changes in the transcriptome after masitinib treatment

**Figure 3:**
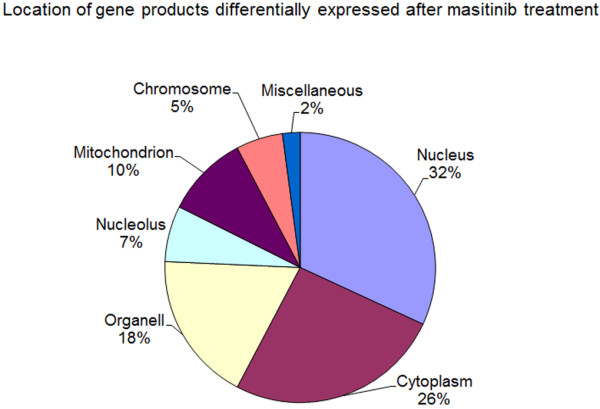
**Microarray analysis of transcriptional changes after masitinib treatment.** Masitinib treated cells showed substantial changes in the global gene expression pattern with up to 3,500 genes or approximately 16% of the canine genome having a significant change in transcription activity.

**Figure 4:**
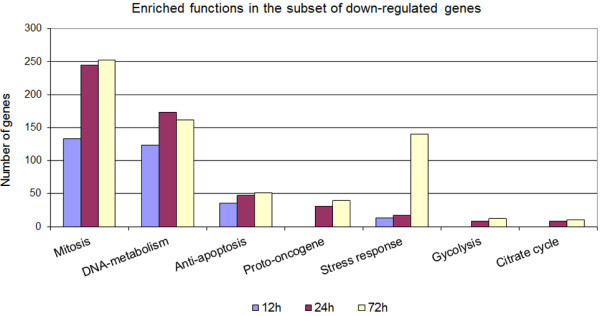
Cellular location of gene products differentially expressed after masitinib treatment.

Treatment of C2 cells with masitinib (100nM) induced a massive change in their global gene expression pattern. A total of 2,116, 3,087 and 3,502 genes had significant changes in their expression levels of > 1.5-fold after 12, 24 and 72 h, respectively (Figure 3, Additional file 1). Approximately 59% of these genes had decreased expression levels while the rest had increased expression. Approximately one third of these genes code for nuclear proteins while 18–26% of the gene products are expressed in the cytoplasm and in cell organelles (Figure 4).

**Figure 5:**
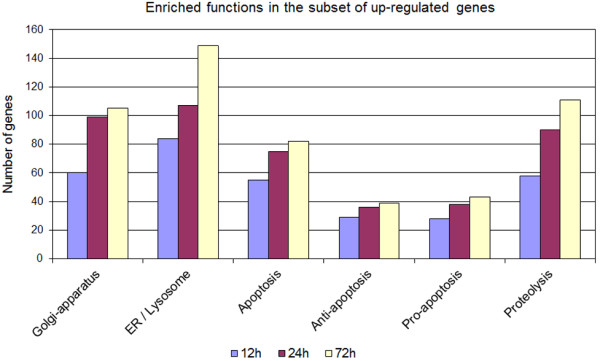
**Gene functions enriched in the subset of down-regulated genes.** Genes associated with functions in cell division, anti-apoptosis, stress response, carbohydrate metabolism were significantly enriched in the subset of down-regulated genes after masitinib treatment.

Most nuclear factors were involved in mitosis and DNA replication (Figure 5), which were mostly down-regulated after masitinib treatment. In addition, genes associated with stress response, glycolysis and the citrate cycle were significantly down-regulated (Figure 5).

**Figure 6:**
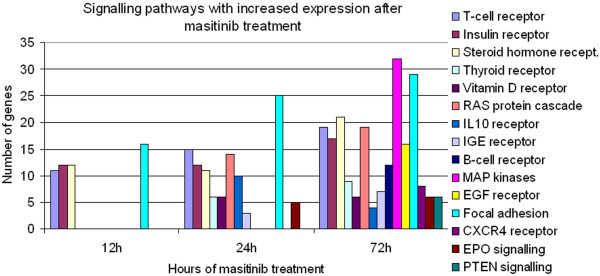
**Gene functions enriched in the subset of up-regulated genes.** Genes associated with functions in the Golgi-apparatus, the endoplasmic reticulum and apoptosis were significantly enriched in the subset of up-regulated genes after masitinib treatment.

An up-regulation of mRNA expression levels was mostly observed for genes associated with Golgi apparatus, endoplasmic reticulum and lysosomes and genes associated with apoptosis and proteolysis (Figure 6). Of note, a set of pro-apoptotic genes were significantly enriched in both, up-regulated and down-regulated, groups of genes.

**Figure 7:**
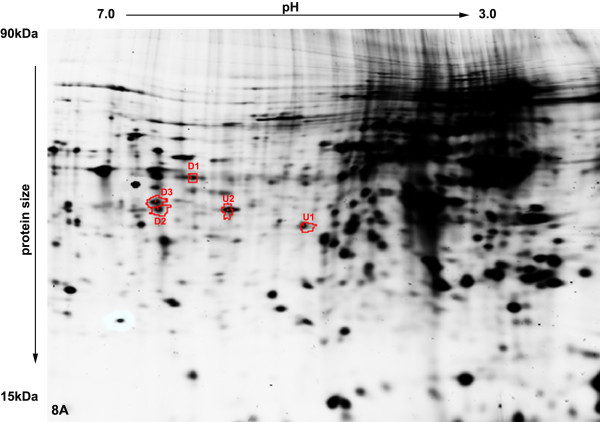
**Potentially KIT dependant pathways.** Several pro-proliferative pathways have up-regulated mRNA expression levels after masitinib treatment and may thus reflect efforts of treated cells to activate alternative proliferation stimuli and circumvent growth arrest and cell death.

Pathway analysis identified a significant down-regulation of gene expression levels associated with p53, steroid receptor and GTPase-associated signal transduction pathways. In contrast, there was a time dependent increase in the number of up-regulated genes associated with signal transduction pathways during masitinib treatment. After 12 hours of masitinib treatment there was a significant up-regulation of genes associated with three signal transduction pathways, i.e. T-cell receptor, insulin receptor and steroid hormone receptor. At 24 hours genes associated with five additional pathways were up-regulated, i.e. thyroid receptor, vitamin D receptor, Ras cascade, IL10 receptor and IGE receptor. Finally, at 72 hours up-regulation of genes associated with 15 signal transduction pathways were recorded, the aforementioned pathways and the signalling cascades of the B-cell receptor, MAP kinase, EGF receptor, focal adhesion, CXCR4 receptor, EPO signalling and PTEN signalling (Figure 7).

A correlation analysis of expression levels with the different timepoints identified 89 genes with a time dependent, continuous up-regulation in gene expression levels during masitinib treatment, including the cyclin-dependent kinase inhibitor 1A (p21, Cip1), parathyroid hormone (PTH) and platelet/endothelial cell adhesion molecule 1 (PECAM1) ( Additional file 2). DAVID analysis identified a significant enrichment of the functional annotations apoptosis, ATM-signalling pathway, RAS protein signal transduction, aging, B-cell proliferation and unfolded protein response in this group of genes. A correlation analysis identified 55 genes that had a time dependent, continuous decrease in expression levels during masitinib treatment, including EIF2 and EIF5. Enriched functional annotations in this gene subset were butyrate and pyruvate metabolism, mitochondrial functions, cell migration, apoptosis and mitosis ( Additional file 2).

### Changes in the proteome after masitinib treatment

**Figure 8:**
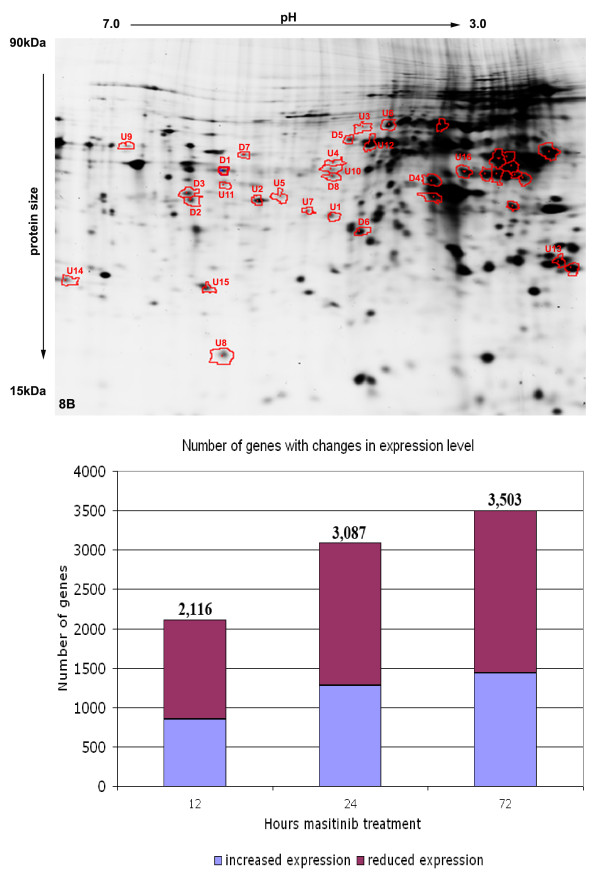
**Differentially expressed proteins in masitinib treated C2 cells after 24 (A) and 72 hours (B) when compared to untreated cells.** D1–8: proteins down-regulated in treated cells; U1–U25 (red): proteins up-regulated in treated cells. Two-dimensional difference gel electrophoresis of cell culture lysates. Isoelectric focusing pH 3–7, gel width 24 cm.

2D-DIGE and MALDI-TOF analyses of the proteome after 24 and 72 hours of masitinib treatment identified 24 proteins with significant differences in protein expression levels when compared to the proteome before masitinib treatment (Figure 8A and 8B). Three proteins, TAR-DNA binding protein (TARDBP, 1.52-fold, *p* = 0.012), eukaryotic translation initiation factor 3 (EIF3, 1.30-fold, *p* = 0.014) and the actin related protein 2 (ACTR 2, 1.09-fold, *p* = 0.0054) were down-regulated after 24 hours of masitinib treatment (Table 1). Only two proteins, annexin A1 (ANXA1, 1.66-fold, *p* = 0.0087) and the gelsolin-like capping protein (CAPG, 1.66-fold, *p* = 0.0039) were up-regulated after 24 hours of masitinib treatment.

**Table 1:** Down- or up-regulated proteins after 24 hours masitinib treatment

**Spot**	**Identified protein**	**AV**	**p-value**	**accession no. in NCBI**	**MW [Da]**^§^	**pI**^§^	**MOWSE score**	**assigned peptides**	**sequ. cov. [%]**
*Down-regulated proteins*									
1	TARDBP	−1.52	0.012	gi|6678271 (Ho)	45053	5.85	53	1*	4*
2	EIF3	−1.30	0.014	gi|73974369 (Ca)	29504	7.78	92	8	21
3	ACTR2	−1.09	0.0054	gi|73969816 (Ca)	36488	8.21	46	1*	4*
Up-regulated proteins									
4	ANXA1	+1.66	0.0087	gi|73946797 (Ca)	38887	5.84	91	12	31
5	CAPG	+1.66	0.0039	gi|73980918 (Ca)	39088	6.04	85	6	24

^§^The listed molecular weights and pI values correspond to the listed accession numbers, which sometimes belong to species other than *Canis familiaris* (Ca.).

*These proteins were identified by unique peptide sequences determined by MALDI-TOF MS/MS experiments.

Abbreviations: *EIF3*: eukaryotic translation initiation factor 3; *ACTR2*: actin-related protein 2; *ANXA1*: annexin *A1*; *MCP*: Macrophage capping protein; *Ca*: Canis familiaris; *Ho*: Homo sapiens.

The effect of masitinib treatment on all five proteins was confirmed by comparing the proteome at 72 hours of treatment with the pre-treatment proteome. All five proteins were identified as significantly regulated at 24 hours and having an even increased expression level after 72 hours treatment (Figure 8B, Table 2). Nineteen additional proteins had significant changes in expression levels after 72 hours treatment (Table 2). Proteins with the highest down-regulation were the eukaryotic translation initiation factor 4a (EIF4A, 1.66-fold, *p* = 0.005), T-complex protein 1 alpha (TCP1A, 1.63-fold, *p* = 0.019) and the inorganic pyrophosphatase 1 (PPA1, 1.25-fold, *p* = 0.021). In addition to the two proteins with increased expression levels after 24 hours, 14 up-regulated proteins were identified after 72 hours of masitinib treatment. Of these, iroquois homeobox 6 (IRX6, 1.74-fold, p = 0.0018), selenium binding protein 1 (SELENBP1, 1.65-fold, *p* = 0.0011), ubiquitin carboxyl-terminal esterase L3 (UCHL3, 1.51-fold, *p* = 0.027) and annexin A6 (ANXA6, 1.50-fold, *p* = 0.031) had the highest up-regulation in protein expression levels.

**Table 2:** Down- or up-regulated proteins after 72 hours masitinib treatment

**Spot.**	**Identified protein**	**AV**	**t test**	**accession no. in NCBI**	**MW [Da]**^2^	**pI**^§^	**MOWSE score**	**assigned peptides**	**sequ. cov. [%]**
Down-regulated proteins									
1	TARDBP43	−2.62	0.019	gi|6678271 (Ho)	45053	5.85	53	1*	4*
6	EIF4A	−1.66	0.005	gi|73966207 (Ca)	44692	5.40	89	15	41
7	TCP1A	−1.63	0.019	gi|73945755 (Ca)	60943	5.71	80	9	17
2	EIF3	−1.33	0.012	gi|73974369 (Ca)	29504	7.78	92	8	21
8	PPA1	−1.25	0.021	gi|73953384 (Ca)	33372	5.80	34	1*	2*
9	CCT2	−1.25	0.042	gi|73968673 (Ca)	57737	6.01	80	8	17
3	ACTR2	−1.12	0.019	gi|73969816 (Ca)	36488	8.21	46	1*	4*
10	PSMD6	−1.06	0.040	gi|73981961 (Ca)	99123	9.57	72	10	12
Up-regulated proteins									
11	HSP90B1	+1.18	0.048	gi|50979116 (Ca)	22925	6.23	74	5	24
11	GSTT3	+1.20	0.0077	gi|73995693 (Ca)	27793	6.67	98	8	33
12	YWHAZ.	+1.22	0.019	gi|73974186 (Ca)	26438	5.00	156	17	52
13	PDIA3	+1.26	0.012	gi|74000349 (Ca)	76474	8.95	161	21	30
14	CORO1A	+1.34	0.017	gi|73958506 (Ca)	41955	7.10	52	1*	2*
15	TTC38	+1.34	0.016	gi|73968897 (Ca)	52886	5.67	79	6	12
16	OXCT1	+1.43	0.028	gi|73954281 (Ca)	65275	7.92	90	10	18
17	SEMG1	+1.45	0.017	gi|134093092 (Go)	52065	8.96	83	8	19
18	UROD	+1.45	0.016	gi|73977144 (Ca)	35096	5.50	105	9	29
19	ANXA6	+1.50	0.031	gi|73953627 (Ca)	76241	5.47	207	23	32
20	UCHL3	+1.51	0.027	gi|158749588 (Ra)	26278	5.01	74	5	23
21	SELENBP1	+1.65	0.0011	gi|73981582 (Ca)	67142	7.59	89	9	20
22	IRX6	+1.74	0.0018	gi|121484129 (Pa)	24682	8.99	74	5	24
5	CAPG	+1.98	0.0036	gi|73980918 (Ca)	39088	6.04	85	6	24
4	ANXA1	+2.58	0.007	gi|73946797 (Ca)	38887	5.84	91	12	31

^§^The listed molecular weights and pI values correspond to the listed accession numbers, which sometimes belong to species other than *Canis familiaris* (Ca).

*These proteins were identified by unique peptide sequences determined by MALDI-TOF MS/MS experiments.

Abbreviations: EIF4A: eukaryotic translation initiation factor 4A; TCP-1-alpha: T-complex protein 1, alpha subunit; EIF3: eukaryotic translation initiation factor 3; ACTR2: actin-related protein 2; EIF3: eukaryotic translation initiation factor 3; HSP-ß1: heat shock protein beta-1; PDI: protein disulfide isomerase-associated 3 precursor; CORO-1A: coronin-1A; UPD: Uroporphyrinogen decarboxylase; ANXA6: annexin A6; MCP: Macrophage capping protein; ANXA1: annexin A1; Ca: Canis familiaris; Ho: Homo sapiens; Pan: Pan troglodytes; Ra: Rattus; Go: Gorilla.

Comparison with the set of genes identified in the transcriptome analysis identified 15 gene products to be present in the list of mRNA and proteins with significant changes in expression levels. mRNA expressions from 6 of the 8 down-regulated proteins after masitinib treatment were also down-regulated. Furthermore, mRNA from 9 of the 15 proteins up-regulated in C2 treated cells was also present in the transcriptome analysis. However, only five of the transcripts were up-regulated whereas four were down-regulated, in contrast to the situation at the protein level.

## Discussion

The present study aimed at identifying the transcriptional and translational responses of KIT-mutant canine mast cells after treatment with the TKI masitinib. To this end, C2 cells, a cell line with a tandem duplication in the juxtamembrane unit and thus constitutively activated KIT, were treated with masitinib and changes in the global mRNA and protein expression levels were characterised. Due to the strong dependency of neoplastic mast cell proliferation on the constitutively activated KIT it was hypothesized that the observed effects may directly or indirectly be caused by the inhibition of KIT [1,13].

Treatment of C2 cells with masitinib resulted to a significant change in mRNA expression levels of a substantial number of genes. More than 3,500 genes had up-regulated mRNA expression levels after 72 hours of masitinib treatment. This gene number corresponds to approximately 16% of the suspected 22,000 genes in the canine genome [18]. According to estimations in human cells, approximately 4,000 genes or 16% of the complete coding genome is active in a given cell on average [19], indicating that almost the complete set of active genes in the C2 cells responds to masitinib treatment. This, however, is only a very rough estimation since the number of active genes may certainly be different in the analysed neoplastic mast cells.

The initial observation of reduced proliferation and metabolism of masitinib treated cells lead to the hypothesis that masitinib treatment and thus KIT inhibition causes a severe shut off of gene activity in treated cells. The results of the transcriptome analysis however indicate that almost half of the regulated genes were transcriptionally up-regulated. Relatively few of these genes had a time dependent up- or down-regulation after masitinib treatment as indicated by the analysis of a potential correlation of the changes in gene expression levels and increasing treatment times.

The active transcriptional response of C2 cells to masitinib treatment is in accordance with the observation that there was only a mild increase in LDH release and thus cell death in the medium of masitinib treated cells even after 72 hours. C2 cells therefore seem to actively respond to KIT inhibition with an alternative quantitative and qualitative gene expression pattern to circumvent cell death. For instance, 15 receptor pathways were up-regulated after 72 hours of masitinib treatment, most of which have a potential pro-proliferative activity. It can thus be hypothesised that those pathways may contain potential targets for combination therapy.

A subsequent proteome analysis identified 24 proteins with significant changes in protein expression after 72 hours masitinib treatment. 65% of the proteins also had significant changes in the mRNA expression levels. The total number of proteins is therefore surprisingly low when compared to the large number of transcripts affected. One of the reasons may be a delayed response of the proteome to the changes in the transcriptome and changes in mRNA expression levels may therefore not be reflected after 72 hours [20]. Another point may be the complete metabolic shut off of treated cells which may also severely hamper the protein metabolism of the cells. miRNA as potent regulators of mRNA translation efficiency may also have influenced the differences in mRNA and protein expression levels. On the other hand, two-dimensional difference gel electrophoresis is known to cover only a fraction of the complete proteome while hydrophobic proteins, e.g. membrane proteins and strongly acidic and basic proteins are difficult to separate and visualise by gel electrophoresis. In addition, the differences in the dynamic range of protein and mRNA detection methods also significantly differ and influence the number and intensity of detected mRNA and protein species [21].

## Conclusions

In conclusion, masitinib treatment of neoplastic mast cells leads to a massive change in the global mRNA expression pattern while only few proteins had significant changes in expression levels after three days of treatment. In contrast to our initial hypothesis, a surprisingly high number of genes had an up-regulated expression, indicating cellular efforts to replace KIT activity and circumvent growth arrest by activation of alternative pro-proliferative pathways. However, as is evident from the long-term follow-up study of masitinib treatment in dogs with non-resectable MCT, development of such alternative pathways are by no means guaranteed [14]. Nonetheless, if present in vivo these pathways may contain potential candidates to be identified as targets for a combined therapy with masitinib to further improve the efficiency of mast cell therapy.

## Methods

### Cell line

C2 cells were kindly provided by Patrice Dubeuil (Institut national de la santé et de la recherche médicale (INSERM), Marseille, France). Cells were cultured in RPMI-1640 with stable glutamine medium (Biowest, NuaillÃ©, France) supplemented with 10% FCS (Biowest, NuaillÃ©, France), sodium pyruvate (Biochrome, Berlin, Germany), MEM non-essential Amino Acids (Biowest, NuaillÃ©, France) and penicilline/streptomycine (Gibco, Darmstadt, Germany) and kept at 5% CO2 and 37°C. Cells were passaged every 6 to 7 days and rethawed from an original stock every 10 to 12 weeks. In all experiments, cells from passages 3 through 10 were used. On time point 0 cell culture flasks were incubated with 100 nM masitinib Mesylate (AB Science, Paris, France) and kept at 5% CO2 and 37°C. Three replicates of RNA and protein were obtained at time point 0 before and after 12, 24, 48 and 72 hours of masitinib incubation. The supernatant of each flask was centrifuged at 500 g for 5 minutes and washed twice with un-supplemented RPMI-1640 medium (Biowest, NuaillÃ©, France) with centrifugation steps at 1,000 g for 1 minute. Finally the cell pellet of each flask was equally divided and used for protein or mRNA isolation, respectively. Cell pellets were resolved in 250 μl protein lysis buffer (GE Healthcare, Freiburg, Germany) or 500 μl RA1 lysis buffer (NucleoSpin RNA; Macherey & Nagel, DÃ¼ren, Germany) as previously described. Samples were stored at −80°C until further use. Cell numbers were determined at each time point using Trypan Blue exclusion (Biochrome, Berlin, Germany) and viable cells were counted in a Neubauer microscope counting chamber. The experiments with the C2 cell line were performed according to the European, German and local ethical guidelines of the Freie Universitaet Berlin. Animals, humans and their tissues were not otherwise involved in the study presented here.

### WST-1 assay

For WST and LDH assays, cells were seeded at a density of 1.8 × 10^4^ cells / ml in 96-well plates (Greiner, Frickenhausen, Germany). Mitochondrial activity was quantified using the Cell Proliferation Reagent WST-1 (Roche Diagnostics GmbH, Mannheim, Germany). Following the indicated treatments of cells, 10 μl WST-1 reagent was added per well (1:10 final dilution). After a 1 hour incubation at 37°C, the absorbance at 450/630 nm was measured by using an ELISA reader (Bio-Rad Laboratories GmbH, München, Germany).

### Lactate dehydrogenase release

Lactate dehydrogenase (LDH) activity was determined by using the CytoTox-ONETM Homogeneous Membrane Integrity Assay (Promega GmbH, Mannheim, Germany), a fluorimetric method for measuring the release of LDH from cells with damaged membranes. All reagents were prepared according to the manufacturer’s instructions. CytoTox-ONETM reagent (100 μl) was added following treatments to each well and incubated for a further 10 minutes. Next, 50 μl stop solution was added, the plate was shaken for 10 seconds, and the fluorescent signal was recorded at the 560/590nm excitation/emission wavelength pair by using Fluostar Optima (BMG Labtech GmbH, Offenburg, Germany). Sample triplicates were treated with 5 μl lysis solution to perform a 100% cell lysis control in order to determine the maximum amount of LDH.

### Protein and mRNA isolation

Proteins were extracted in 250 μl protein lysis buffer containing 7 M urea, 2 M thiourea, and 4% CHAPS (3-(3-Cholamidopropyl)dimethylammonio-1-propanesulfonate). Lysates were sonicated twice for 2 minutes and then centrifuged at 2,200 g for 2 minutes. The supernatant was collected and stored at −80°C until analysis. Protein concentrations were determined with the 2-D Quant Kit (GE Healthcare, Freiburg, Germany). For mRNA isolation pellets were transferred into 500 μl of RA1 lysis buffer (NucleoSpin RNA; Macherey & Nagel, DÃ¼ren, Germany) containing 5 μl β-mercaptoethanol and homogenized by pipetting. mRNA was extracted and purified using a commercial system (NucleoSpin RNA; Macherey & Nagel, DÃ¼ren, Germany) [22,23].

RNA quality was controlled using the BioAnalyzer (Agilent Technologies, USA) and only high quality RNA (RIN > 9) was used for microarray analyses.

### Microarray data analysis

Affymetrix GeneChip hybridization (Canine Genome 2.0 Array) was performed with 2 μg total RNA according to the manufacturer’s recommendations. Three chips for each timepoint of treatment and pretreated cells were stained and washed with the GeneChip Fluidics Station 450 and visualized on an Affymetrix GeneChip Scanner 3000. Microarray data were deposited at the Gene Expression Omnibus data repository under the number GSE32657.

Affymetrix CEL files were imported into Partek Genomic Suite Software (Version 6.4, Partek Inc., St. Louis, USA) and processed by the implemented gcRMA workflow (median polish probe set summarization, RMA background correction, quantile normalization) [24]. Differences in gene expression between samples at the different time points of masitinib treatment were analysed by ANOVA and false discovery rate was controlled by using the q-value method [25]. Differentially expressed genes were selected by applying a filter of q < 0.001 and a fold-change of >1.5 in both directions. Un-named genes were excluded from the list. Hierarchical clustering of the samples and genes was conducted using Pearson correlation and complete linkage. Change in expression levels were correlated with the different timpoints and a partial correlation >0.95 was accepted as linear correlated.

To supplement the gene annotations of differentially expressed genes with functional information, BLAST search and Affymetrix-provided human to canine microarray comparisons were used to map canine genes to their human equivalents as shown before [26,27]. Using the human equivalents as templates, the DAVID database was queried for gene ontology information [28]. To study enriched functional gene families and functional annotation, all down-regulated and all up-regulated genes were submitted separately to DAVID [29]. In the case of redundant probes with a fold-change in the same direction only the probe set with the highest fold-change was included in further analyses. Selection criteria for DAVID included a medium stringency, ≥ 4 probes within a cluster and an enrichment factor > 1.3. In case of multiple appearances of similar gene families or functional annotation terms, the cluster with the higher enrichment factor was selected.

### 2D-DIGE and MALDI-TOF

Two-dimensional difference gel electrophoresis (2D-DIGE) was used to quantify and compare the proteome in triplicates of C2 cell pellets before and after 24 and 72 hours of masitinib treatment. Protein extracts were labelled with CyDyes (GE Healthcare, Freiburg, Germany) as previously described [30]. The internal standard was composed of equal amounts of all protein lysates used. 50 μg of protein of the respective samples were labelled with 400 pmol of the respective dye. Two cell pellet probes and the internal standard were then combined and an equal volume of 2× sample buffer (7 M urea, 2 M thiourea, 4% CHAPS, 2% Pharmalyte IPG Buffer, 2% DTT, 0.04% bromophenol blue) was added. Rehydration buffer (7 M urea, 2 M thiourea, 4% CHAPS, 2% pharmalyte IPG buffer, 40 mM DTT) was used to yield a final volume of 450 μl. The Cy-labelled samples were applied to immobilised non-linear pH gradient (IPG) strips, pH 3–7 (GE Healthcare, Freiburg, Germany), and strips were allowed to rehydrate in the dark at room temperature overnight. Isoelectric focusing (IEF) was performed using an Ettan IPGphor 3 Isoelectric Focusing Unit (Ettan IPGphor Manifold; GE Healthcare, Freiburg, Germany) for a total of 50 kVh at 20°C, 75 μA/strip.

Two steps of equilibration followed IEF: 15 minutes with equilibration buffer (6 M urea, 30% glycerol, 2% SDS, and 50 mM Tris, 0.02% bromophenol blue, pH 8.8) containing 100 mg DTT, followed by 15 minutes with equilibration buffer containing 250 mg iodoacetamide. Strips were transferred on top of 24 cm width, 12.5% SDS-PAGE gels and sealed with 0.5% low-melting-point agarose. The second-dimension molecular weight separation was carried out using an Ettan DALTsix Electrophoresis Unit (GE Healthcare, Freiburg, Germany). Running parameters used were 60 mA for 1 hour, 240 mA for 1 hour and 300 mA for 5 hours [31].

CyDye-stained protein spots were visualised with a Typhoon 9400 fluorescence scanner (GE Healthcare, Freiburg, Germany) at the respective wavelengths for the three CyDyes. Spot detection, matching and quantification of spot intensity were performed using the DeCyder 2D Software, Version 7.0 (GE Healthcare, Freiburg, Germany). Differences in expression between the different durations of masitinib treatment were analysed using an unpaired student’s *t* test with *p*-values < 0.05 considered significant. No multiple testing or FDR adjustment was done. Gels with 350 μg of protein were silver-stained and spots were picked for subsequent MS analysis [32].

### Protein identification by MALDI-TOF-MS

For protein identification by matrix-assisted laser desorption/ionisation time-of-flight mass spectrometry (MALDI-TOF-MS) an Ultraflex-II TOF/TOF instrument (Bruker Daltonics, Bremen, Germany) equipped with a Smart beam™ laser was used. Peptides were obtained by trypsin in-gel digestion as previously described [32]. Protein digests were measured in the reflector mode using α-cyano-4-hydroxycinnamic acid (CHCA) as matrix. For the database search, listed contamination peaks from keratin and autoproteolytic products were excluded for peptide mass fingerprint database search with the Mascot server ( http://www.matrixscience.com) in the NCBInr database. The search was restricted to mammalian sequences and one missed tryptic cleavage was considered. A mass accuracy of 50−100 ppm was used for the searches.

## Competing interests

All authors declare to have no financial or non-financial competing interests.

## Authors’ contribution

RK conceived, organized, coordinated the study and experiments, and drafted the manuscript. RK, DL, MH carried out the microarray experiments and analyzed the data. AM and RK carried out the cell culture experiments. AM and AB carried out the WST and LDH assays. PS, AB, RE performed the 2D-DIGE experiments. PS and CW carried out the mass spectrometry. ADG helped to conceive the study and to draft the manuscript. All authors read and approved the final manuscript.

## Supplementary Material

Additional file 1 List of genes with significant changes in mRNA expression levels after 12h, 24 and 72 hours of masitinib treatment. Click here for file

Additional file 2 Genes with a time-dependent, continuous increase or decrease in mRNA expression level during masitinib treatment. Click here for file
